# Choked: A Case Report of Oculopharyngeal Muscular Dystrophy Mimicking Hypothyroidism From the Philippines

**DOI:** 10.7759/cureus.41025

**Published:** 2023-06-27

**Authors:** Jerome M Infante, Belinda Lioba Nepomuceno

**Affiliations:** 1 Institute for Neurosciences, Section of Neurology, St. Luke’s Medical Center, Quezon City, PHL

**Keywords:** philippines, dysphagia, ptosis, hypothyroidism, oculopharyngeal muscular dystrophy

## Abstract

Oculopharyngeal muscular dystrophy (OPMD) is a late-onset myopathic genetic disorder characterized by chronic progressive dysphagia and ptosis with or without proximal limb weakness. It is most often caused by an abnormal alanine-encoding (GCN) trinucleotide repeat expansion in the first exon of the poly(A)-binding protein nuclear 1 (*PABPN1)* gene. Patients with hypothyroidism may similarly report bilateral ptosis, dysphagia, and limb weakness. Here, we report the case of a 65-year-old Austrian female with hypothyroidism living in the Philippines who presented with gradually progressive ptosis, dysphagia, and intermittent choking episodes. No known relatives had similar symptoms. Physical examination showed bilateral symmetric ptosis, good cough reflex, and good limb muscle strength. Electromyographic studies of facial and laryngeal muscles were found to be normal. Thyroid evaluation showed biochemically hyperthyroid status while taking Levothyroxine. With a clinical suspicion for genetic progressive myopathy, we considered OPMD. Genetic testing revealed abnormal expansion of GCN trinucleotide repeats in the *PABPN1* gene. We describe the first reported case of OPMD with detected *PABPN1* gene expansion in the Philippines simulating hypothyroidism symptoms, suggesting possible points for misidentification and underdiagnosis of OPMD in developing countries.

## Introduction

Oculopharyngeal muscular dystrophy (OPMD) is a rare late-onset genetic myopathy beginning in adulthood in the fifth to sixth decade and is characterized by gradually progressive ptosis, dysphagia, and/or proximal limb weakness [1]. It is predominantly caused by an expansion from the normal 10 alanine-encoding (GCN) trinucleotide repeats in the first exon of the poly(A)-binding protein nuclear 1 (*PABPN1) *gene to an abnormal 11-18 GCN repeats in 90% of OPMD patients [2]. Other genetic findings identified in OPMD cases include biallelic GCN trinucleotide repeat expansion and c.35G > C point mutation in the *PABPN1* gene [2-4]. The highest prevalence has been reported among Bukhara Jews in Israel (1:600) [5] and among French-Canadians in Quebec (1:1,000) [6], while the prevalence in European countries has been reported to be 1:100,000-1,000,000 [7]. A handful of case reports have been published across Asia [8], with no published reports from the Philippines.

Hypothyroidism, a common endocrinologic disorder among females, can manifest with neuromuscular symptoms. Proximal limb weakness has been reported in 25% of cases [9]. Ptosis has been associated with hypothyroidism, although rare and often mild [10,11]. Dysphagia is another infrequent symptom of hypothyroidism but has been reported in severe cases with frequent choking [12,13].

Here, we report a case of OPMD diagnosed in the Philippines presenting with chronic progressive bilateral ptosis, dysphagia, and choking, which was misidentified as a manifestation of hypothyroidism. This report aims to increase awareness of this rare genetic progressive myopathy for early detection, management, and counseling.

## Case presentation

A 65-year-old Austrian woman living in the Philippines was admitted for aspiration pneumonia following episodes of choking. She had a history of difficulty in swallowing solid foods with intermittent choking episodes for the last 20 years. She began experiencing drooping of bilateral upper eyelids 12 years ago. At the same time, she was diagnosed with primary hypothyroidism and was prescribed Levothyroxine with good compliance. In the interim, she had worsening bilateral eyelid drooping, persistent dysphagia to solids, and intermittent choking, with two episodes requiring a Heimlich maneuver. Multiple consultations with ophthalmologists and neurologists were unrevealing. Her symptoms were attributed to hypothyroidism. She did not have any known relatives from Austria with similar symptoms. Paternal family history was not available from her estranged father. The patient was subsequently referred for evaluation of swallowing while being treated for pneumonia.

A physical examination of the patient revealed bilateral symmetric ptosis, intact gag reflex, absent dysarthria, and good facial and limb muscle strength. A fiberoptic endoscopic evaluation of swallowing showed diffuse pooling of salivary secretions with penetration but no actual aspiration of secretions. Food testing showed pharyngeal residue with puree, mashed fruits, and gelatin intake. There were occasional penetration instances on puree but no actual aspiration. The thyroid evaluation revealed the patient to be clinically euthyroid and biochemically hyperthyroid, with low serum thyroid-stimulating hormone (TSH) and elevated serum triiodothyronine (FT3) and thyroxine (FT4) while on Levothyroxine therapy. Creatine kinase muscle isoform level was normal (Table 1). Single-fiber electromyography, electromyography (EMG), and motor conduction studies of the face, larynx, and upper extremities were normal (Tables 2-4). The laryngeal ultrasound was unremarkable.

**Table 1:** Serum tests performed for myopathy workup.

Test	Results	Reference value
Creatine phosphokinase total	101 U/L	26–192
Creatine kinase-MM	89 U/L	21–215
Free triiodothyronine	5.05 pg/mL	2.30–4.20
Free thyroxine	2.20 ng /dL	0.89–1.76
Thyroid-stimulating hormone	0.01 µlU/mL	0.55–4.78

**Table 2:** Electromyography of the facial, laryngeal, and upper extremity muscles and single-fiber electromyography. Stimulation of the facial, laryngeal, and upper extremity muscles evoked well-defined compound muscle action potentials bilaterally with normal insertional activity and absent spontaneous activity. Single-fiber electromyography showed jitter values within normal limits for age.

Muscles		Insertional activity	Spontaneous activity			Motor unit potential	Single-fiber electromyography – mean jitter (µs)
			Fibrillation	Fasciculation	Positive sharp waves		
Facial muscles	Left frontalis	Normal	-	-	-	Normal form, amplitude, and duration. Normal recruitment pattern	
	Right frontalis	Normal	-	-	-	Normal form, amplitude, and duration. Normal recruitment pattern	
	Left orbicularis oris	Normal	-	-	-	Normal form, amplitude, and duration. Normal recruitment pattern	25.3 µs
	Right orbicularis oris	Normal	-	-	-	Normal form, amplitude, and duration. Normal recruitment pattern	
Laryngeal muscles	Left thyroarytenoid	Normal	-	-	-	Normal form, amplitude, and duration. Normal recruitment pattern	
	Right thyroarytenoid	Normal	-	-	-	Normal form, amplitude, and duration. Normal recruitment pattern	
	Left cricothyroid	Normal	-	-	-	Normal form, amplitude, and duration. Normal recruitment pattern	
	Right cricothyroid	Normal	-	-	-	Normal form, amplitude, and duration. Normal recruitment pattern	
Upper extremity muscles	Left deltoid	Normal	-	-	-	Normal form, amplitude, and duration. Normal recruitment pattern	
	Left biceps	Normal	-	-	-	Normal form, amplitude, and duration. Normal recruitment pattern	
	Left flexor carpi radialis	Normal	-	-	-	Normal form, amplitude, and duration. Normal recruitment pattern	
	Left extensor digitorum communis	Normal	-	-	-	Normal form, amplitude, and duration. Normal recruitment pattern	
	Left first dorsal interosseous	Normal	-	-	-	Normal form, amplitude, and duration. Normal recruitment pattern	

**Table 3:** Facial nerve stimulation study. Stimulation of the facial nerves with simultaneous recording over at orbicularis oculi, frontalis, and nasalis muscles showed normal compound muscle action potential amplitudes and distal latencies with no significant side-to-side differences.

Muscle	Parameter	Left	Right
Orbicularis oculi	Distal latency	4.1 ms	3.0 ms
	Amplitude	0.3 mV	0.8 mV
Nasalis	Distal latency	4.6 ms	4.5 ms
	Amplitude	0.8 mV	0.8 mV
Frontalis	Distal latency	3.8 ms	3.9 ms
	Amplitude	0.4 mV	0.8 mV

**Table 4:** Motor conduction study of the upper extremities. Motor conduction studies performed on the left and right median and ulnar nerves showed normal compound muscle action potential amplitudes, distal motor latencies, and conduction velocities.

Nerve	Site	Amplitude	Conduction velocity	Distal latency
Left median nerve	Wrist	10.5 mV	53 m/s	3.3 ms
	Elbow	10.1 mV		
Right median nerve	Wrist	6.3 mV	57 m/s	3.3 ms
	Elbow	6.0 mV		
Left ulnar nerve	Forearm	11.3 mV	56 m/s	2.7 ms
	Elbow	10.9 mV	53 m/s	
	Above elbow	10.7 mV		
Right ulnar nerve	Forearm	7.5 mV	58 m/s	2.9 ms
	Elbow	7.0 mV	54 m/s	
	Above elbow	6.8 mV		

With a clinical suspicion for a chronic progressive late-onset myopathic disease, we considered a genetic etiology, particularly OPMD. Gene testing revealed abnormal repeat expansion of the *PABPN1 *gene to 14 trinucleotide repeats, consistent with a genetic diagnosis of an autosomal dominant OPMD.

## Discussion

We described a hypothyroid female patient with OPMD, misidentified as having a neuromuscular manifestation of hypothyroidism. Both OPMD and hypothyroidism can manifest with ptosis and dysphagia, although in hypothyroidism, the said symptoms are usually milder and unusual [10-14]. Ptosis in hypothyroidism has been described to be bilateral, intermittent, and can be asymmetric [10,11], while dysphagia was associated with choking and dysphonia [12-14]. The more common neuromuscular manifestations of hypothyroidism include myalgias, elevated creatine kinase levels, and proximal myopathy commonly affecting the shoulder and hip girdle muscles. These are usually in conjunction with systemic hypothyroidism symptoms such as weight gain, cold intolerance, and dry skin [11,12]. These neuromuscular manifestations of hypothyroidism respond significantly to thyroid replacement therapy [10-14]. Our patient had persistent, bilateral, and symmetric ptosis with dysphagia and choking that did not improve with Levothyroxine therapy.

OPMD is characterized by worsening ptosis, dysphagia, and proximal limb weakness. Ptosis has been reported to be the most consistent symptom found in almost all OPMD cases, while 62-100% of patients had dysphagia and 20-81% presented with limb weakness [8]. The mean age of onset has been reported to be 48 years for ptosis and 50 years for dysphagia [2]. Those with symptom onset before 45 years of age develop severe OPMD associated with proximal lower limb weakness by the age of 60 [2]. The pattern of inheritance often described in OPMD is autosomal dominant, but cases have shown a recessive inheritance pattern [2,8]. Our patient initially presented with dysphagia with intermittent choking at 45 years and then symmetric progressive ptosis at 53 years without concomitant limb weakness. The patient had no known familial history of muscular disorders or of similar signs, although paternal family history was unknown. With a high clinical suspicion for genetic muscular pathology and the nonconformity of symptoms to hypothyroidism and other common causes of ptosis including myasthenia gravis, we performed a genetic testing which revealed an autosomal dominant OPMD diagnosis.

Definitive diagnosis of OPMD is established through genetic testing identifying a trinucleotide repeat expansion in the *PABPN1 *gene. A delay of three to 20 years from the onset has been reported in OPMD owing to its gradual progression of symptoms and mimicking other diseases including myasthenia gravis [15]. Literature has shown muscle ultrasound can be a potential biomarker for detecting muscle changes in OPMD [16]. Management remains to be supportive with no curative interventions yet. Blepharoplasty can be considered for severe ptosis. For dysphagia, dietary modification and swallowing therapy should be initiated. For severe dysphagia at risk of fatal choking, cricopharyngeal dilatation, botulinum toxin injection, and myotomy can be considered [17]. Routine surveillance of neuromuscular signs and family genetic counseling are also crucial in the management of OPMD. The patient and her family were appraised of the diagnosis and were advised symptom monitoring, swallowing therapy, and counseling.

This paper reports an Austrian-born female genetically confirmed to have OPMD, the first reported diagnosed case of OPMD misidentified as hypothyroidism from the Philippines. Owing to its late onset and the nonspecific symptoms of dysphagia and ptosis, which may simulate neurologic manifestations of other illnesses, OPMD may be misidentified as another disease entity and is likely underdiagnosed, particularly in developing countries like the Philippines. The majority of reported and published OPMD cases have been contributed by Western countries with over 80% of cases, while the more developed countries such as Japan and Taiwan contributed more to the already limited number of reports among Asian countries [18]. As of the time of writing, there are no published local reports and data on OPMD cases from the Philippines. Diagnosis can be challenging as genetic testing is not widely available and is limited in most developing countries [18], often opting to send out samples abroad. Reasons for the underdiagnosis of OPMD in developing countries may include unawareness, unfamiliarity, or failure to recognize OPMD as a differential diagnosis by doctors in patients presenting with ptosis and dysphagia, as well as the lack of available, easily accessible, and economical gene testing centers to confirm the clinical suspicion of OPMD.

## Conclusions

Clinicians should consider OPMD for patients presenting with chronic progressive dysphagia and bilateral ptosis after an extensive workup for common causes and if with clinical suspicion for genetic etiology. OPMD remains underdiagnosed and often misidentified, particularly in developing countries. Increasing awareness of OPMD can improve diagnosis, allowing early appropriate intervention and genetic counseling.
